# Flocculation Efficiency and Mechanism of Carbamazepine by Microbial Flocculant Extracted from *Klebsiella pneumoniae* J1

**DOI:** 10.1155/2020/8811516

**Published:** 2020-11-18

**Authors:** Jie Xing, Nanzhe Song, Xiangwei Chen, Ang Li, Hongwei Ni

**Affiliations:** ^1^Northeast Forestry University, Harbin 150040, China; ^2^Heilongjiang Provincial Research Academy of Environmental Sciences, Harbin 150056, China; ^3^Institute of Natural Resources and Ecology, HAS, Harbin 150040, China; ^4^State Key Laboratory of Urban Water Resource and Environment, School of Environment, Harbin Institute of Technology, Harbin 150090, China

## Abstract

The microbial flocculant (MFX) extracted from *Klebsiella pneumoniae* J1 was used to remove carbamazepine in prepared wastewater and domestic sewage. The influence factors and flocculation mechanism were studied. The optimal carbamazepine removal conditions for MFX were pH of 7-8, 7 mL of flocculant, 0.1 mL of coagulant, and 35°C, and the removal rate reached 81.75%. MFX was efficient in the removal of carbamazepine in both domestic sewage (75.03%) and secondary sedimentation tank effluent (69.76%). The pseudo-first-order kinetic equation fitted the adsorption process better than the pseudo-second-order kinetic equation, which suggested that the adsorption was not pure chemical adsorption. The analysis of floc size suggested that the repulsive force between carbamazepine and MFX was weakened under alkalescent conditions, which can help the growth and coherence of flocs and increase the carbamazepine removal efficiency. Enough dosage of MFX can generate larger flocs, but excessive dosage of MFX will decrease the carbamazepine removal rate because of increase in electrostatic repulsion. The analysis of 3D-EEM and FTIR suggested that hydroxyl, amino, and carboxyl in MFX played an important role in the removal of carbamazepine. As an eco-friendly and highly efficient microbial flocculant, MFX has potential for practical applications in carbamazepine removal.

## 1. Introduction

Carbamazepine (CBZ), a typical pharmaceutical and personal care product (PPCP) [1–3], is a medicine for the treatment of epilepsy, depression, glossopharyngeal neuralgia, trigeminal neuralgia, and central partial diabetes insipidus [4]. Toxicological experiments show that the LD50 of carbamazepine is 4205 mg/kg (for rats). Carbamazepine has been detected in sewage treatment stations since 1998, and it has been found that the concentration of carbamazepine in water is higher than that of other PPCPs in recent years [5]. Carbamazepine widely exists in the effluent of urban sewage plants, surface water, and soil. Carbamazepine may eventually enter drinking water and groundwater through environmental migration and transformation [3, 6].

It can be seen from the pollution status of carbamazepine in the environment that carbamazepine widely exists in various environmental media after migration and transformation, and it has good stability and durability, allowing it to exist in the environment for a long time [7, 8]. Therefore, it is urgent to improve the removal efficiency of carbamazepine in the research of PPCP pollutants.

Microbial flocculants (MFX), extracted from microorganisms, widely exist and are nontoxic and eco-friendly [9–11]. There are numerous chemical groups (e.g. hydroxyl, carboxyl, and amino) in MFX, which allow MFX to have good combining capacity with pollutants and have good flocculation efficiency [12–15]. In addition to the removal of turbidity of wastewater, it has been proved that MFX can treat wastewaters that contain heavy metals [16–18] and antibiotics [19–21]. Because there is no secondary pollution of MFX, it is one of the most promising materials to treat wastewater [22].

Until now, most studies on MFX used prepared solution to study the removal efficiency of MFX. Actually, the constituents in actual wastewater are much more complex than those in a prepared solution [23]. There are many other contaminants in actual wastewater, and these contaminants could compete against carbamazepine, which will decrease the carbamazepine removal efficiency by MFX. Therefore, besides prepared solutions, it is also important to conduct experiments on carbamazepine removal in actual wastewater.

In this study, MFX was used to remove carbamazepine from wastewater. The carbamazepine removal efficiency was tested under different conditions (pH, dosage of MFX, dosage of coagulant, and temperature). Considering the practical application of MFX, the carbamazepine removal efficiency by MFX was also studied in domestic sewage and in secondary sedimentation tank effluent. The adsorption kinetics and the mechanism of carbamazepine flocculation were studied.

## 2. Materials and Methods

### 2.1. Materials and Reagents

The extraction of MFX from *Klebsiella pneumoniae* J1 (CGMCC No. 6243) was described in our previous work [19]. Briefly, J1 was inoculated and fermented to produce MFX. MFX was extracted by alcohol extraction and was purified by dialysis in ultrapure water. Dried MFX powder was obtained by vacuum freeze drying and stored at 4°C.

Carbamazepine, CaCl_2_, and the reagents used to prepare MFX were purchased from Bailingwei Technology Co., Ltd. Ultrapure water (18 M*Ω*/cm, Milli-Q), and analytical-grade reagents were used in this work.

### 2.2. Batch Adsorption Experiments

The stock carbamazepine solution (5 mg/L) was prepared by dissolving carbamazepine in ultrapure water and stored at 4°C for later use. The stock carbamazepine solutions were diluted appropriately to obtain the experimental solutions. MFX (1 g/L) and coagulant aid (CaCl_2_, 10%) were added to the initial carbamazepine solution (1 mg/L, 1 L). After flocculating and stirring for a period of time, the samples were allowed to stand for 20 min. After adsorption, MFX was separated by 0.45 *μ*m filter membrane and the concentration of carbamazepine was determined by high-performance liquid chromatography (HPLC, Shimadzu, LC-10A (SPD/RID/RFD), Japan). All experimental data were the average of triplicate measurements. The adsorption capacity (*q*_*e*_, mg/g) of carbamazepine by MFX was calculated as follows:

(1) $$\begin{array}{c} q_{e}=\left(C_{0}–C_{e}\right)\frac{V}{M}, \end{array}$$

where *C*_0_ and *C*_*e*_ are the initial and equilibrium carbamazepine concentrations (mg/L), respectively, *V* is the volume of the carbamazepine operating solution (L), and *M* is the dose of MFX (g).

MFX was also used to adsorb carbamazepine in domestic sewage and sewage from the secondary settling tank from the Harbin Taiping Sewage Treatment Plant. The adsorption process was similar to that of carbamazepine solution. The difference was that the concentration of carbamazepine in the domestic sewage and the sewage from the secondary settling tank was much lower than the prepared carbamazepine solution; therefore, the samples were pretreated via ethyl acetate liquid-liquid extraction. To be specific, the same volume of ethyl acetate was added to the filtered supernatant, the sample was shaken for 10 min, and then, it was allowed to stand for layering. The supernatant liquid was extracted, shaken for 5 min, and then allowed to stand for layering. The supernatant liquid was extracted. After rotary evaporation and nitrogen blowing of the collected upper liquid, it was redissolved with chromatographic grade methanol. The concentration of carbamazepine was measured by high-performance liquid chromatography (HPLC, Waters, USA). Three parallel samples were prepared for each sample. Carbamazepine (20 *μ*g/L) was used as a standard sample to measure the recovery rate of the spiked standard.

### 2.3. Adsorption Kinetics

The adsorption kinetics of MFX for carbamazepine were discussed using the pseudo-first-order and pseudo-second-order kinetic models. The initial concentration of carbamazepine was at a pH of 7, with CaCl_2_ (0.1 mL, 10%) as a coagulant aid. The adsorption times were 5, 10, 15, 20, 30, 40, 50, and 60 min, respectively. The pseudo-first-order and pseudo-second-order kinetic models are displayed in

(2) $$\begin{array}{c} \log\left(q_{e}-q_{t}\right)=\log\left(q_{e}\right)-\frac{k_{1}}{2.303}t,\\ \frac{t}{q_{t}}=\frac{1}{k_{2}{q_{e}}^{2}}+\frac{t}{q_{e}}, \end{array}$$

where *q*_*e*_ and *q*_*t*_ are the equilibrium adsorption capacity and adsorption capacity at a certain moment, respectively (mg/g), *t* is the adsorption time (min), and *k*_1_ and *k*_2_ are the rate constants of pseudo-first-order and pseudo-second-order kinetic models, respectively.

### 2.4. Analysis of Mechanisms of Carbamazepine Adsorption on MFX

A granulometer (Malvern MS2000, UK) was used to monitor the growth of different microbial flocculants and to calculate the growth rate of the flocs in the adsorption process. The functional groups were determined by Fourier transform infrared spectroscopy (FTIR, Perkin-Elmer spectrum 100). Three-dimensional fluorescence spectrophotometry (3D-EEM, FP6500, JASCO, Japan) was used to obtain EEM spectra, and the fluorescence response was obtained by scanning the emission spectra from 220 to 650 nm and by varying the excitation wavelength from 220 to 500 nm. Fluorescence quenching, completed by adding carbamazepine into the MFX solution, was used to assess the adsorption characteristics.

## 3. Results and Discussion

### 3.1. Factors That Influence the Removal Efficiency of Carbamazepine via MFX

Generally, to evaluate the adsorption capacity of MFX, the removal efficiency of a pollutant needs to be tested under different conditions. In this study, the adsorption performance of carbamazepine by MFX was studied under different conditions by changing the pH, dosage of MFX, dosage of coagulant aid, and temperature, respectively.

#### 3.1.1. pH

**Figure 1:**
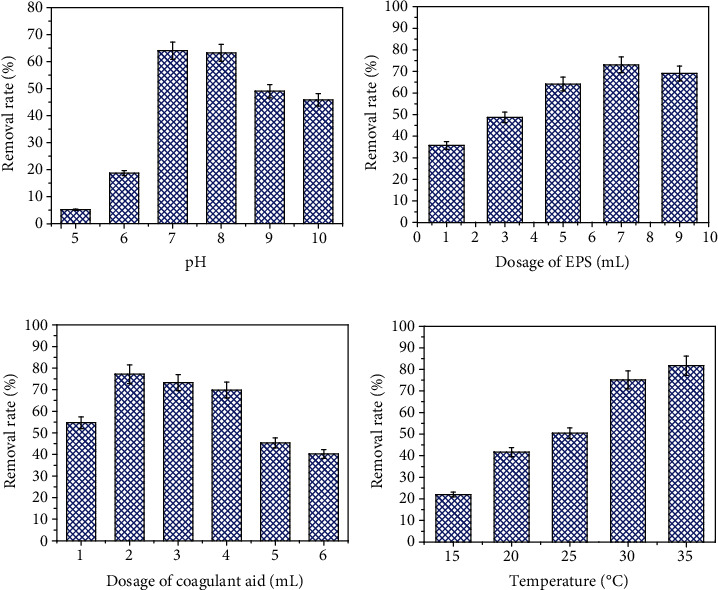
Removal efficiency of carbamazepine on MFX at different conditions: (a) pH, (b) dosage of MFX, (c) dosage of coagulant aid, and (d) temperature.

The pH value is an important parameter in the adsorption process. The pH not only affects the functional groups of MFX but also affects the surface charge of carbamazepine [24, 25]. The removal rate of carbamazepine by MFX was tested at pH = 5–10, and the results are shown in Figure 1(a). It was obvious that the removal rate of carbamazepine by MFX increased from pH = 5 to pH = 7 and decreased from pH = 7 to pH = 10. MFX showed a poor carbamazepine removal effect under acidic conditions. At pH = 5, the removal rate was only 5.14%. However, the carbamazepine removal rate became better under nonacidic conditions. At pH = 7, MFX had the best carbamazepine removal rate (65.26%), and the removal rate decreased apparently at pH = 9–10. This suggests that both acidic and overly alkaline conditions are not suitable for the removal of carbamazepine by MFX. This was because the change of pH conditions changed the quantity and properties of surface charges of both MFX and carbamazepine, and overly acidic and overly alkaline conditions can weaken the neutralization effect and hinder the aggregation reaction between MFX and carbamazepine [20]. Therefore, the optimal pH range for the removal of carbamazepine by MFX is 7–8.

#### 3.1.2. Dosage of MFX

The dosage of MFX is another important factor that can affect the removal rate of carbamazepine [26]. The removal rate of carbamazepine by MFX was tested after adding a different dosage of MFX, and the results are shown in Figure 1(b). The carbamazepine removal rate increased when the dosage increased from 1 mL to 7 mL but then decreased with the increase of dosage of MFX. MFX performed the best when its dosage was 7 mL and the maximum removal rate was 73.06%. When 9 mL dosage of MFX was added to carbamazepine, the removal rate showed a downward trend. This was because when the dosage of MFX was low, the quantity of MFX was not enough to remove carbamazepine even if MFX reached adsorption saturation. However, when the dosage was too high, the excessive MFX could influence the charge property of the entire system and destroy the balance of charge, which subsequently decreased the carbamazepine removal rate. This result was similar to those in other studies [27].

#### 3.1.3. Dosage of Coagulant Aid

Coagulant aids can improve the flocculation conditions by adjusting the charge property of the system [21]. In this study, CaCl_2_ was added as a coagulant aid, and the carbamazepine removal rate of MFX with different dosage of coagulant aid is shown in Figure 1(c). The CaCl_2_ dosages of samples 1 to 6 were 0 mL, 0.1 mL, 0.3 mL, 0.5 mL, 1 mL, and 1.5 mL, respectively. It can be seen that the carbamazepine removal rate by MFX was 54.73% when no coagulant was added, which proves that MFX can remove carbamazepine alone. When the dosage was 0.1 mL, the carbamazepine removal rate increased and reached the maximum removal rate of 77.17%. However, with the increase of CaCl_2_ dosage, the carbamazepine removal rate decreased gradually. This was because carbamazepine was removed by MFX through adsorption bridging between macromolecules, and the coagulant aid can strengthen the adsorption bridging by adjusting the charge property of the system. However, excessive dosage of the coagulant aid will introduce excessive positive charges, which bind to the negatively charged adsorption sites in MFX, hindering the connection of carbamazepine and MFX. Therefore, the optimal dosage of coagulant aid needs to be determined by adsorption experiments. In this study, the optimal dosage was 0.1 mL.

#### 3.1.4. Temperature

Temperature is another important factor that can affect the carbamazepine removal efficiency by MFX [26].  Figure 1(d) shows the carbamazepine removal rate by MFX at 15°C, 20°C, 25°C, 30°C, and 35°C, respectively. It can be seen that the removal rate increased as the temperature increased. The removal rate was only 22.06% at 15°C, but increased to 81.75% at 35°C. Generally, an appropriate temperature increase can accelerate the random movement between molecules, which then increase the collision probability among molecules and increase the carbamazepine removal rate by MFX. However, too high of a temperature will lead to the inactivation of MFX, which will decrease the carbamazepine removal rate [28]. In this study, MFX performed the best at 35°C, suggesting MFX was effective even at 35°C.

In summary, the optimal carbamazepine removal conditions for MFX were pH 7-8, 7 mL of flocculant, 0.1 mL of coagulant, and 35°C. The removal rate reached 81.75%.

### 3.2. Carbamazepine Removal from Domestic Sewage

**Figure 2:**
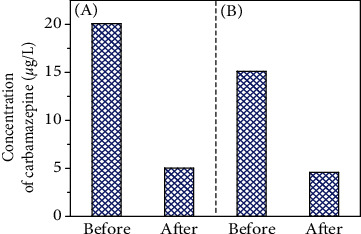
Removal efficiency of carbamazepine on MFX: (a) domestic sewage and (b) secondary sedimentation tank effluent.

The above experiments prove that MFX has a good carbamazepine removal efficiency for a prepared carbamazepine solution (1 mg/L). However, the carbamazepine concentration in domestic sewage is usually tens to hundreds of *μ*g/L. Moreover, the constituents in domestic sewage are much more complex than a prepared carbamazepine solution [23]. Although domestic sewage contains a large number of suspended solid colloidal particles that are conducive to the formation of flocs, the competition for the adsorption sites on MFX between carbamazepine and other constituents can lead to the decrease of carbamazepine removal rate. Figures 2(a) and 2(b) show the removal rate in domestic sewage and in secondary sedimentation tank effluent. It can be seen that MFX was efficient in the removal of carbamazepine both in domestic sewage (75.03%) and in secondary sedimentation tank effluent (69.76%), which suggested that MFX had a good removal effect on carbamazepine.

### 3.3. Adsorption Kinetics

Adsorption kinetics is important when studying adsorption mechanism. The kinetics analyzes the effects of adsorption time on the adsorption of pollutants by describing the adsorption rate and then explores the adsorption mechanism. Usually, the first-order, second-order, pseudo-first-order, and pseudo-second-order kinetic equations are used to analyze the adsorption kinetics. In this study, MFX and carbamazepine were reactants in the adsorption process. Based on the characteristics of MFX and the applicable scope of each kinetic equation, the pseudo-first-order and pseudo-second-order kinetic equations were selected to fit the experimental data in this study.

In the first-order kinetics, the reaction rate is only proportional to the concentration of one reactant, while in the pseudo-first-order kinetics, the reaction rate is related to the concentration of the adsorbent and the adsorbate. When the concentration of one substance is much more than the other, it shows the characteristics of a first-order kinetic reaction [29, 30].

**Figure 3:**
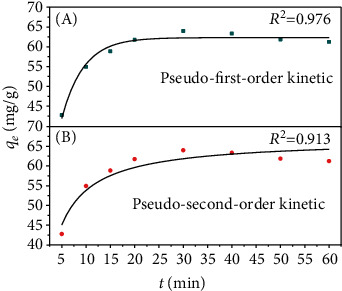
The fitting curve of the (a) pseudo-first-order kinetics and (b) pseudo-second-order kinetics.

Figures 3(a) and 3(b) show the fitting results of the pseudo-first-order and pseudo-second-order kinetic equations.  Table 1 shows the parameters of these kinetic equations. In Figure 3(a), the adsorption reaction reached adsorption equilibrium in 20 min, and the maximum adsorption capacity was 62.32 mg of carbamazepine per gram of MFX. In the actual experiment, the maximum adsorption capacity was 62.99 mg/g and the adsorption time was 30 min, which were consistent with the values predicted by the pseudo-first-order kinetic equation. In Figure 3(b), the predicted adsorption did not reach adsorption equilibrium in 60 min, and the predicted maximum adsorption capacity (66.89 mg/g, 60 min) was more than the actual value. Moreover, the *R*^2^ of the pseudo-first-order kinetic equation is 0.976, which is much higher than the *R*^2^ of the pseudo-second-order kinetic equation. These results suggested that the adsorption of carbamazepine by MFX was more in line with the pseudo-first-order kinetic equation.

According to other studies, the adsorption process described by the first-order kinetic is controlled by substance transport. The pseudo-second-order kinetic simulates the second-order kinetic, which describes a chemical reaction, accompanied by electron sharing or transfer. The adsorption of carbamazepine by the MFX-fitted pseudo-first-order kinetic better than the pseudo-second-order kinetic, which suggested that the adsorption was not pure chemical adsorption [17].

### 3.4. Mechanisms of Carbamazepine Adsorption on MFX

**Figure 4:**
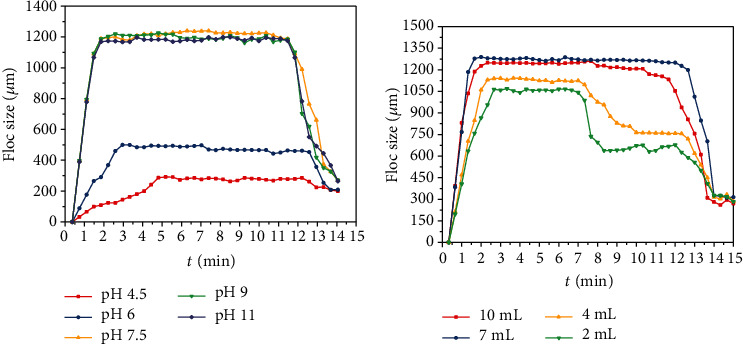
The effect of factors on floc formation: (a) pH and (b) dosage of MFX.

The preliminary experimental results showed that the pH, dosage of MFX, dosage of coagulant, and temperature can affect the carbamazepine removal rate by MFX. The dosage of MFX and pH were the main factors; therefore, the flocculation morphology was used to further investigate the carbamazepine removal mechanism by MFX. The changes of floc size under different pH values/dosages of MFX at different times are shown in Figure 4. The flocs under different pH conditions showed a similar changing trend in the whole coagulation processes. The floc formation process was slow under acidic condition, and the particle size was relatively small in the stable stage. Moreover, there was no obvious sedimentation at pH = 4.5. The floc formation process was the fastest at pH = 7.5. When the pH was larger than 7.5, the growth of the flocs was significantly accelerated, the settling velocity of the flocs was fast, and the changes were not obvious as the pH became larger. This was because the surface charge density of MFX and carbamazepine was low under an alkalescent condition, which can weaken the repulsive force between carbamazepine and MFX and can help the growth and coherence of flocs [31].

In Figure 4(b), the maximum floc size was the smallest when the dosage of MFX was 2 mL (1064 *μ*m), and the floc size became larger with the increase of MFX dosage. However, when the dosage was 10 mL (1235 *μ*m), the floc size became smaller than that under a dosage of 7 mL (1288 *μ*m). Moreover, the growth of the floc was slower at a low dosage of MFX, and the maintenance time of the stable period was shorter, which was not conductive to the removal of carbamazepine. This was because too low of a dosage of MFX cannot provide sufficient adsorption sites for carbamazepine, and the collision probability between MFX and carbamazepine was low. Therefore, enough dosage of MFX can generate larger flocs. However, the dynamic equilibrium period was short with too high dosage of a MFX, because there was a large amount of the same charge with excess MFX, which increased electrostatic repulsion. Similar phenomena have been found in the study of the coagulation of microbial flocculant and kaolin, and this was caused by the adsorption bridging effect.

**Figure 5:**
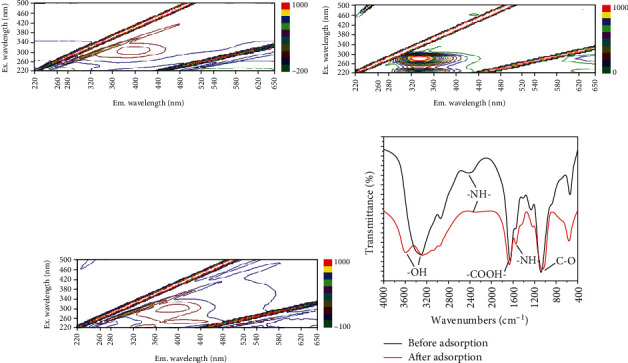
EEM of component of MFX adsorbing carbamazepine: (a) carbamazepine aqueous solution, (b) precipitation after adsorption, (c) liquid supernatant after adsorption, and (d) FTIR of MFX before and after carbamazepine.

To investigate whether there was chemical reaction between carbamazepine and MFX, 3D-EEM and FTIR were used to analyze the samples before and after adsorption. Figures 5(a)–5(c) show the EEM of carbamazepine aqueous solution, precipitation after adsorption of carbamazepine by MFX, and liquid supernatant after adsorption, respectively. In Figure 5(a), it can be seen that the carbamazepine aqueous solution only had an absorption peak at *λ*ex/*λ*em = (310‐320)nm/(395‐405)nm. This peak belongs to Class I (humic acid-like area), which was caused by typical exogenous organics. In this study, this peak was caused by carbamazepine [32]. In Figure 5(b), the precipitate after adsorption shows two high concentration absorption peaks at *λ*ex/*λ*em = (270‐280)nm/(325‐335)nm and *λ*ex/*λ*em = (225‐235)nm/(325‐335)nm, which are the characteristic peaks of proteins that are the main active components of MFX [20]. No characteristic peak of carbamazepine was found in this spectrum, because carbamazepine has been adsorbed on MFX through intermolecular forces and it was not a free molecule that can be detected. In Figure 5(c), three obvious absorption peaks can be seen in the spectrum of the supernatant after adsorption. The peak at *λ*ex/*λ*em = (310‐320)nm/(395‐405)nm is the characteristic peak of carbamazepine. The peak at *λ*ex/*λ*em = (225‐235)nm/(325‐335)nm belongs to the tyrosine and benzene ring structure protein related to biological sources, which represent a small amount of the active ingredients of MFX or its derivatives. The peak related to tryptophan at *λ*ex/*λ*em = (250‐280)nm/(325‐335)nm disappeared, and a new peak resembling fulvic acid at *λ*ex/*λ*em = (250‐270)nm/(410 − 420)nm appeared. This peak was a new peak after MFX adsorbed carbamazepine, which suggested that chemical reactions occurred between carbamazepine and the tryptophan-like component on MFX.

To further prove that chemical reactions occurred between carbamazepine and MFX, FTIR was used to analyze MFX before and after adsorption of carbamazepine (Figure 5(d)). Compared to the FTIR spectrum of MFX before adsorption, there was a new peak in the FTIR spectrum of MFX after adsorption, accompanied by multipeak displacement. The new peak appeared at 3590.22 cm^−1^, which was the characteristic peak corresponding to hydroxyl. The absorption peak caused by the stretching vibration of associative hydroxyl at 3309.94 cm^−1^ exhibited a redshift to 3291.88 cm^−1^, and the peak shape became wider. This proved that the hydroxyl played a role in the adsorption of carbamazepine by MFX, and this was achieved by hydrogen bonding. The absorption peak caused by the vibration of amino at 2435.06 cm^−1^ exhibited a redshift to 2321.08 cm^−1^, and the intensity was significantly weakened. The long and narrow absorption peak at 1654.45 cm^−1^ was a characteristic peak of carboxyl, and this peak exhibited a blueshift to 1669.71 cm^−1^. The peak at 1549.75 cm^−1^ caused by the bending vibration of amide exhibited a redshift to 1546.45 cm^−1^, which proved that the active amino in the proteins of MFX participated in the adsorption reaction. The long and narrow absorption peak at 1080.57 cm^−1^ as a typical absorption peak of sugar derivatives included the stretching and vibration of C-O and C-O-C. The peak exhibited a redshift to 1047.34 cm^−1^ after adsorption of carbamazepine. These results proved that the hydroxyl, amino, and carboxyl groups were all involved in the adsorption reaction and played a role together in the removal of carbamazepine by MFX [17].

## 4. Conclusion

MFX was used to remove carbamazepine. MFX was efficient in the removal of carbamazepine in both domestic sewage and secondary sedimentation tank effluent. The pseudo-first-order kinetic equation can describe the adsorption process. The adsorption was a compound process that involved physical and chemical adsorption. MFX performed the best under alkalescent conditions, because the repulsive force between carbamazepine and MFX was weakened, which can help the growth and coherence of flocs. Enough dosage of MFX can generate larger flocs, but excessive dosage of MFX will decrease the carbamazepine removal efficiency due to the increase of electrostatic repulsion. The hydroxyl, amino, and carboxyl groups in MFX played an important role in the removal of carbamazepine. As a widely existing, nontoxic, and eco-friendly microbial flocculant, MFX has the potential for practical applications in carbamazepine removal from domestic sewage, carbamazepine wastewater, and natural water bodies.

## Figures and Tables

**Table 1:** The parameters of the pseudo-first-order and pseudo-second-order kinetics.

Equation	Experimental *q*_*e*_ (mg/g)	Predicted *q*_*e*_ (mg/g)	*k*_1_	*R* ^2^
Pseudo-first-order kinetic	62.99	62.32	-0.22	0.98
Pseudo-second-order kinetic	62.99	66.89	0.0062	0.91

## Data Availability

The data that support the findings of this study are available from the corresponding author Jie Xing, upon reasonable request.
